# High-Pressure Acceleration of Nanoliter Droplets in the Gas Phase in a Microchannel

**DOI:** 10.3390/mi7080142

**Published:** 2016-08-15

**Authors:** Yutaka Kazoe, Ippei Yamashiro, Kazuma Mawatari, Takehiko Kitamori

**Affiliations:** 1 Department of Hemolysis and Apheresis, Graduate School of Medicine, The University of Tokyo, 7-3-1 Hongo, Bunkyo, Tokyo 113-8656, Japan; kazoe@icl.t.u-tokyo.ac.jp; 2 Deparment of Applied Chemistry, Graduate School of Engineering, The University of Tokyo, 7-3-1 Hongo, Bunkyo, Tokyo 113-8656, Japan; yamashiro@icl.t.u-tokyo.ac.jp (I.Y.); kmawatari@icl.t.u-tokyo.ac.jp (K.M.)

**Keywords:** microfluidics, microchannel, droplet, gas phase

## Abstract

Microfluidics has been used to perform various chemical operations for pL–nL volumes of samples, such as mixing, reaction and separation, by exploiting diffusion, viscous forces, and surface tension, which are dominant in spaces with dimensions on the micrometer scale. To further develop this field, we previously developed a novel microfluidic device, termed a microdroplet collider, which exploits spatially and temporally localized kinetic energy. This device accelerates a microdroplet in the gas phase along a microchannel until it collides with a target. We demonstrated 6000-fold faster mixing compared to mixing by diffusion; however, the droplet acceleration was not optimized, because the experiments were conducted for only one droplet size and at pressures in the 10–100 kPa range. In this study, we investigated the acceleration of a microdroplet using a high-pressure (MPa) control system, in order to achieve higher acceleration and kinetic energy. The motion of the nL droplet was observed using a high-speed complementary metal oxide semiconductor (CMOS) camera. A maximum droplet velocity of ~5 m/s was achieved at a pressure of 1–2 MPa. Despite the higher fluid resistance, longer droplets yielded higher acceleration and kinetic energy, because droplet splitting was a determining factor in the acceleration and using a longer droplet helped prevent it. The results provide design guidelines for achieving higher kinetic energies in the microdroplet collider for various microfluidic applications.

## 1. Introduction

Microfluidics has enabled the fabrication of miniaturized chemical systems, known as lab-on-a-chip and micro-total analysis systems (μTAS), for chemical analysis, medical diagnosis, and chemical synthesis [1,2]. By exploiting diffusion, viscous forces, and surface tension, which are dominant in small spaces due to the short diffusion distance and increased surface-to-volume ratio, effective and fast micro-unit operations (MUOs) such as mixing, reaction, and separation have been developed [3,4,5,6]. Based on these principles, integrated chemical systems have exhibited superior performances, i.e., shorter processing times (from days or hours to minutes or seconds) and smaller sample/reagent volumes (down to μL), compared to conventional bulk chemical systems.

Our group has developed a new microfluidic device, termed a microdroplet collider, which exploits spatially and temporally localized kinetic energy in small spaces [7]. A microdroplet in the gas phase is formed in a microchannel, accelerated, and made to collide with a target. Acceleration to a velocity of ~1 m/s was demonstrated, which is a more than 100 times faster velocity (i.e., 10,000 times higher kinetic energy) than the values achievable by conventional microfluidic transport of a droplet in an oil phase [4,8]. The inelastic and minimally deformable collisions exploited by using the confined spaces with dimensions on the micrometer scale achieved highly efficient energy transfer compared to collisions between droplets in free space, which involve energy dissipation by deformation mechanisms such as bouncing, coalescence, disruption, and fragmentation [9,10]. Compared to mixing by diffusion of similar-sized droplets, the microdroplet collider achieved 6000-fold faster mixing. The higher the droplet acceleration, the higher the kinetic energy and efficiency of the chemical operations performed.

However, the droplet acceleration has not been optimized since the experiments were conducted for only one droplet size and at pressures of 10–100 kPa. Previously, we found that the microdroplet splits at a high velocity, due to wetting of the droplet tail on the channel wall [11], suggesting an upper limit to the droplet acceleration. 

Recently, we have developed a high-pressure (MPa) control system [12], which may allow higher droplet acceleration. In the present study, we investigated the acceleration of microdroplets in the gas phase in a microchannel at a pressure in the order of 1 MPa. We adjusted the droplet length by varying the channel design, and applied pressures in the order of 1 MPa for higher acceleration. The motion of the accelerated droplets was observed using a high-speed complementary metal oxide semiconductor (CMOS) camera. Based on our results, we discuss the effects of droplet size and pressure on the acceleration and kinetic energy of the droplets.

## 2. Experimental Section

**Figure 1:**
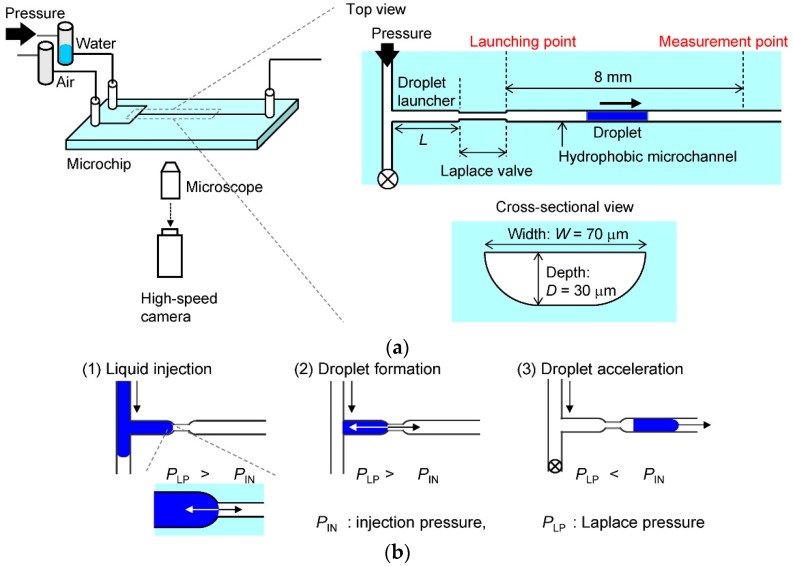
Schematics of (**a**) experimental setup for acceleration of a microdroplet in gas phase in a microchannel and (**b**) microfluidic process for droplet formation and acceleration. The microfluidic device consists of a droplet launcher, a Laplace valve and an acceleration microchannel. The channel wall is modified hydrophobically. During the process, (1) liquid is injected into the droplet launcher, (2) a droplet is formed by air flow, and (3) the droplet is accelerated in the microchannel.

Figure 1 illustrates schematics the experimental setup and microfluidic process used for droplet formation and acceleration. The microchip for the microdroplet collider was connected to a high-pressure (MPa) control system for pressure-driven flow control [12]. The control system was equipped with an inverted microscope, a 2× objective lens, and a high-speed CMOS camera (FASTCAM, Photron, Tokyo, Japan) with a pixel size of 20 μm.

The microchip consisted of a droplet launcher with a width of 70 μm and a depth of 30 μm, a Laplace valve (40*^W^* × 10 μm*^D^*), and an acceleration microchannel (70*^W^* × 30 μm*^D^*). We prepared two microchips with droplet launcher lengths, *L* = 1 and 2 mm. Microchannels with two different depths were fabricated on a glass substrate by two-step photolithographic wet etching, as reported previously [13]. The substrate was thermally bonded with another glass substrate having inlet and outlet holes for gas and liquid injection. The channel wall was modified with an amorphous fluoropolymer (INT-332VE, NI material) to make the surface hydrophobic. The static contact angle measured by a contact angle meter was θ = 117°. Due to the wet etching, the cross-sectional shape of the channel was rounded-rectangular, as illustrated in Figure 1a. We approximated the cross-sectional area of the channel, *A*, and the wetted perimeter, *P*, using the equations *A* ≈ (*W* − 2*D*)*D* + π*D*^2^/2 and *P* ≈ 2*W* − 2*D* + π*D*, respectively, where *W* is the channel width and *D* is the channel depth. Based on these approximations, the hydraulic diameter of the channel, *D*_h_, was 39 μm, as given by *D*_h_ = 4*A*/*P*.

The microfluidic process used for droplet formation and acceleration (Figure 1b) resembled that used in our previous reports [7,11]. A key feature of the process is the Laplace valve [14], which utilizes the Laplace pressure given by the Young-Laplace equation, *P*_LP_ = −4γcosθ/*D*_h_, where γ is the surface tension. The Laplace pressure of the valve was calculated to be *P*_LP_ = 8.4 kPa, using the surface tension at the water–air interface, γ = 72.3 mN/m. First, water was injected into the droplet launcher at a pressure below the Laplace pressure (*P*_IN_ < *P*_LP_). Then, air was injected into the channel to form the microdroplet (*P*_IN_ < *P*_LP_). Finally, a pressure higher than the Laplace pressure was applied to accelerate the droplet in the acceleration channel (*P*_IN_ > *P*_LP_). The volumes of the microdroplet were 1.7 and 3.4 nL, as estimated using the size and length of the droplet launcher.

In this study, the droplet motion was captured using a high-speed CMOS camera at frame rates of 13,333 to 25,000 Hz. The droplet position in the images captured was determined with a spatial resolution of 10 μm, which corresponds to the image pixel size, given by pixel size/magnification. Then, the droplet velocity and droplet length were determined at a measurement position 8 mm away from the launching point.

## 3. Results and Discussion

### 3.1. Acceleration of Microdroplet under MPa-Order Pressure

**Figure 2:**
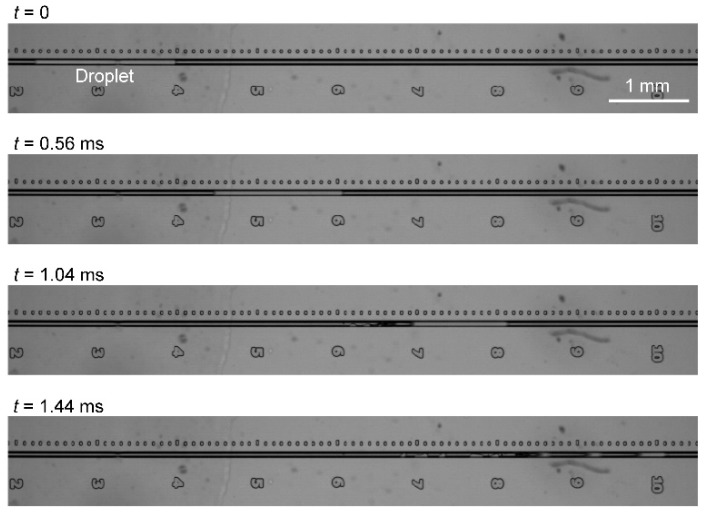
Images of a droplet in a microchannel with *L* = 2.00 mm at an applied pressure of 1800 kPa. *t* = 0 is defined as the time at which the droplet front passes the point 4 mm from the launching point.

Figure 2 shows the motion of an accelerated droplet in a microchannel with *L* = 2.00 mm at an applied pressure of 1800 kPa. A video of the accelerated droplet is presented in the Supplementary Material (Video S1). Within 1.5 ms, the droplet moves through the channel, begins to split from its tail, and disappears. Since the volume of the droplet is only in the order of nL, the influence of water evaporation on the droplet splitting and disappearance must be considered. In order to verify the influence, we estimated the time it would take for the nL droplet to evaporate, based on the evaporation rate of a water droplet on a solid surface reported in a previous study [15]. Assuming that evaporation occurred at the air–water interfacial area in the cross-section of the channel, the time for the evaporation of the nL droplet was estimated to be 10 s. This time scale is much larger than that for the droplet splitting and disappearance in 1 ms as shown in Figure 2. Therefore, the effect of evaporation is considered to be negligible.

Next, the velocity, length, and kinetic energy of the droplet were determined from the captured images. In case of the droplet splitting, we evaluated the length of the first droplet, because it collides with a target in the process. The kinetic energy of the droplet was estimated from 1/2ρ*AL*_D_*U*^2^, where ρ is the density (998 kg/m^3^ for water), *L*_D_ is the droplet length, and *U* is the droplet velocity. We used the cross-sectional area of channel *A* as the cross-sectional area of the droplet, assuming that the cross-section of the channel is filled entirely by the droplet. However, it is still unclear whether the droplet fills a sharp corner of the wet-etched channel (Figure 1a), where the channel wall is modified hydrophobically. Since the channel cross-section seems to be almost entirely filled by the droplet (see Figure 2), which was confirmed in a previous study [11], the dry area in the corner is considered to be small compared to the total area, even if the channel cross-section is not completely filled.

**Figure 3:**
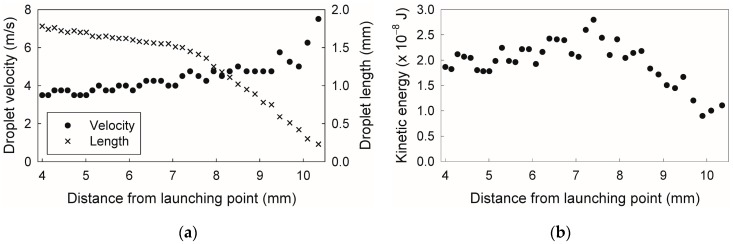
(**a**) Velocity, length, and (**b**) kinetic energy of accelerated droplet as functions of distance from the launching point in a microchannel with *L* = 2.00 mm at an applied pressure of 1800 kPa.

As shown in Figure 3a, when the droplet front passes the point 4 mm from the launching point (*t* = 0), the droplet velocity and length are 3.5 m/s and 1.8 mm, respectively. The velocity increases when the droplet splits, reaching 7.5 m/s just before disappearance, because the fluid resistance decreases in proportion to the droplet length. The fluctuation in the droplet velocity is due to the instability of the wetting, caused in turn by the variation in the dynamic contact angle with splitting, as reported in our previous study [11]. During droplet splitting, the droplet length initially decreases gradually, and no large droplet fragments are observed (*t* = 0 ms and *t* = 0.56 ms in Figure 2). After the droplet passes the 7.5 mm mark, it becomes unstable and rapidly splits with large fragments (*t* = 1.04 ms and *t* = 1.44 ms in Figure 2). This rapid splitting with large fragments occurs when the droplet length falls below a certain value depending on the launcher length and pressure, and often shows variability between experiments conducted under nominally identical conditions. In the rapid splitting state, in addition to the instability of the wetting, Rayleigh instabilities are considered to be a dominant factor in the splitting, because splitting is accompanied by fluctuation of the droplet width, and the droplet splits once the droplet width becomes too small, as shown in Figure 2 and Video S1.

Since the droplet length decreases with splitting, the kinetic energy cannot be at maximum when the droplet velocity reaches a maximum. In the case of the channel with *L* = 2.00 mm at an applied pressure of 1800 kPa, the maximum kinetic energy is attained at a 7.4-mm distance from the launching point, as shown in Figure 3b. At the maximum kinetic energy, the droplet velocity and length were 4.8 m/s and 1.5 mm, respectively.

### 3.2. Relationship between Droplet Acceleration and Applied Pressure

**Figure 4:**
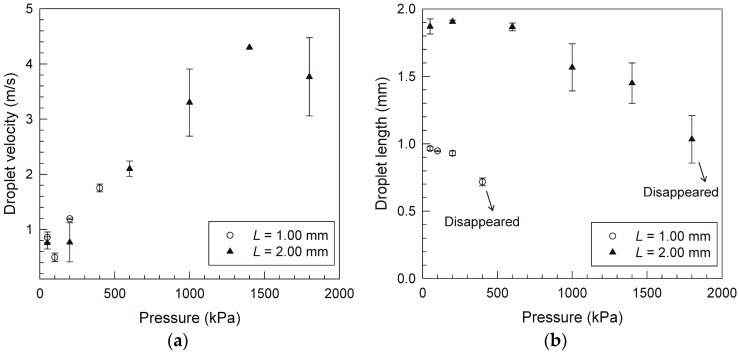
(**a**) Velocity and (**b**) length of droplet as function of applied pressure in microchannels of *L* = 1.00 mm and *L* = 2.00 mm. Error bars represent the standard deviation of triplicate measurements.

Figure 4 shows the droplet velocity and length at a 8-mm distance from the launching point, as a function of the applied pressure. The error bars represent the standard deviation of triplicate measurements. As shown in Figure 4a, for both channels (*L* = 1 and 2 mm), the droplet velocity is around 1 m/s at a pressure below 200 kPa, and increases linearly with increasing pressure above 200 kPa. These results suggest that a longer droplet is more stable without splitting and suitable for higher acceleration. As shown in Figure 4b, the droplet in the *L* = 1 mm channel does not split at 200 kPa, but rather starts to split at higher pressures, and disappears when the pressure exceeds 400 kPa. This is because the droplet velocity increases with increasing pressure as shown in Figure 4a, which leads to the droplet splitting as a result of wetting and Rayleigh instabilities, triggered by an increase in wall friction. The maximum velocity with keeping the initial length is 1.2 m/s, and that with splitting is 1.8 m/s. In the case of the *L* = 2 mm channel, the droplet does not split at 600 kPa, but rather at a higher pressure, and disappears at pressures exceeding 1800 kPa. The maximum velocity with keeping the initial length is 2.1 m/s, and that with splitting is 4.3 m/s. Thus, the maximum velocity achieved in the *L* = 2 mm channel was roughly double that of the *L* = 1 mm channel. The opposite trend is observed in the case of the fluid resistance, which increases in proportion to the droplet length: compared to the *L* = 1 mm channel, the droplet in the *L* = 2 mm channel has twice the fluid resistance, and requires twice the pressure to get a similar velocity. This is because droplet splitting, rather than fluid resistance, is the determining factor in droplet acceleration. Therefore, a design that maintains the droplet stability is most important for high acceleration.

**Figure 5:**
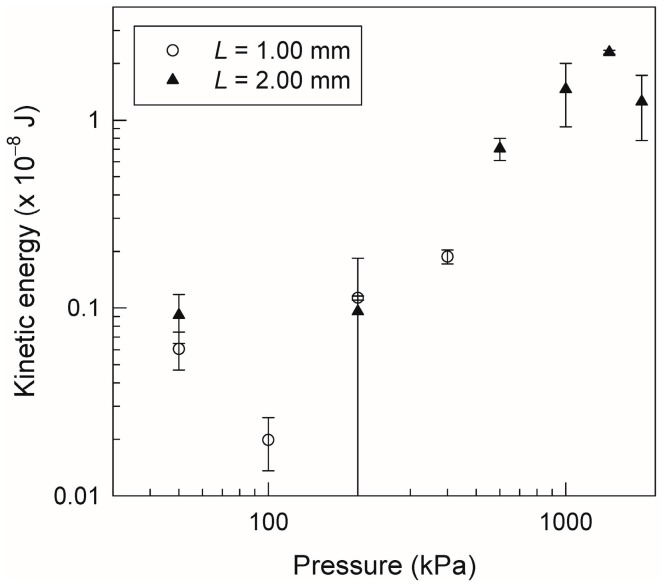
Kinetic energy of the droplet as function of applied pressure in microchannels of *L* = 1.00 mm and *L* = 2.00 mm. Error bars are calculated by propagating the standard deviations for the droplet velocity and length.

Next, we discuss the kinetic energy of the accelerated droplet, which is important for MUOs conducted by the microdroplet collider. Figure 5 shows the kinetic energy calculated from the results as a function of the applied pressure. The error bars are calculated by propagating the standard deviations for the droplet velocity and length. Clearly, longer droplets are more suitable for obtaining higher kinetic energy because of their stability, which prevents splitting. In addition, the kinetic energy achievable by droplet acceleration at 1 MPa is approximately one order of magnitude higher than the value achievable at 10^−1^ MPa, as reported in our previous study [11]. The knowledge obtained from these results provides a design guideline for the microdroplet collider. For example, in order to get the highest possible kinetic energy, multistep acceleration with droplet splitting at a pressure of 1 MPa was effective because of the higher droplet velocity. On the other hand, if a well-controlled droplet volume is required for a given application, acceleration without droplet splitting at 10^−1^ MPa would be more suitable. Thus, this study will contribute greatly to applications using the microdroplet collider, such as enhanced mixing/reactions, injection into continuous flows, and chemical injection into cells and tissues, by exploiting spatially and temporally localized kinetic energy.

Finally, we estimated the level of improvement that can be expected through higher acceleration of the droplet in the case of mixing. In our previous work [11], we observed the mixing process of two droplets by a high-speed CMOS camera combined with an image intensifier. The results suggested that during the mixing, after an accelerated droplet collides with a target droplet, the cusp of the accelerated droplet penetrates the target droplet, maintaining its velocity owing to the effect of inertia. Since the inertial force, with a Reynolds number of 10^1^–10^2^, is dominant, it is thought that convection, which is proportional to the fluid velocity, is a dominant factor in the mixing. By using a pressure in the order of MPa, we achieved a 10-fold increase in droplet velocity relative to our previous study [11]; thus, a 10-fold increase in the speed of mixing can be expected.

## 4. Conclusions

We investigated the acceleration of microdroplets with volumes in the order of nL in the gas phase in microchannels using a high-pressure (MPa) control system. We characterized the droplet motion by using a high-speed CMOS camera to determine the droplet velocity and length. A maximum droplet velocity of ~5 m/s was achieved at a high pressure of 1–2 MPa. The velocities achieved by the microdroplet collider, which are comparable to those achieved by inkjet nozzles [16,17], are 100 times faster than the microfluidic transport of droplets in gas phase reported in a previous study [18], and 10,000 times faster than the velocities obtained when simply sliding a droplet on an inclined surface [19,20]. Despite the higher fluid resistance, longer droplets were more suitable for higher acceleration because droplet splitting, which limits the droplet acceleration, could be prevented by using longer droplets. These results suggested that multistep acceleration with droplet splitting at a pressure of 10^0^ MPa was effective for obtaining the highest kinetic energy. On the other hand, if a given application requires well-controlled droplet volumes, acceleration without droplet splitting at 10^−1^ MPa pressure would be more suitable. The understanding and insight gained through this study will help to establish design guidelines for the microdroplet collider for various microfluidic applications.

## Supplementary Materials

The following are available online at http://www.mdpi.com/2072-666X/7/8/142/s1, Video S1: Accelerated droplet in microchannel of *L* = 2.00 mm at an applied pressure of 1800 kPa (1/2500-speed).
